# Enhancement of adenovirus infection and adenoviral vector-mediated gene delivery by bromodomain inhibitor JQ1

**DOI:** 10.1038/s41598-018-28421-x

**Published:** 2018-08-01

**Authors:** Baojie Lv, Jingjing Li, Meng Li, Yujie Zhuo, Ke Ren, Erguang Li, Guang Yang

**Affiliations:** 1 State Key Laboratory of Pharmaceutical Biotechnology, College of Life Sciences, Nanjing University, Nanjing, China; 2 Nanjing Children’s Hospital, Nanjing Medical University, Nanjing, China; 3 Jiangsu Laboratory of Molecular Medicine, Medical School of Nanjing University, Nanjing, China; 4 School of Laboratory Medicine, Chengdu Medical College, Chengdu, China

## Abstract

Adenovirus-based vectors are among the most commonly used platforms for gene delivery and gene therapy studies. One of the obstacles for potential application is dose-related toxicity. We show here that adenovirus infection and Ad-mediated gene delivery can be enhanced by inhibitors of bromodomain and extra-terminal (BET) family proteins. We showed that JQ1, but not its inactive enantiomer (−)-JQ1, dose-dependently promoted Ad infection and Ad-mediated gene delivery in both epithelial and lymphocyte cells. Given orally, JQ1 also enhanced transgene expression in a murine tumor model. Inhibitors of histone deacetylases (HDACi) are among the commonly reported small molecule compounds which enhance Ad-mediated gene delivery. We found that JQ1 treatment did not cause histone acetylation nor expression of Ad attachment receptor CAR. Instead, JQ1 treatment induced an increase in BRD4 association with CDK9, a subunit of P-TEFb of transcription elongation. Concurrently, we showed that CDK9 inhibition blocked Ad infection and JQ1 enhancement on the infection. The study exemplifies the potentials of BET inhibitors like JQ1 in oncolytic virotherapy.

## Introduction

Adenovirus-based vectors are among the most commonly utilized platforms for gene delivery in cell biology studies and in gene therapy applications. Adenoviral vectors are advantageous in that their abilities to achieve a high efficiency of transduction, high levels of gene expression, and to infect non-dividing cells. Ad vectors have been studied extensively in tumor therapy trials^1,2^. In addition to the use of a conditionally-replicative Ad vector for head and neck cancer in China^3^, recent approval of an oncolytic herpesvirus-based treatment by the US Food and Drug Administration (FDA) raises optimism on the potential of viral vectors in cancer therapy^4^.

Significant hurdles exist that prevent the realization of clinic application of Ad vectors. This is primarily because of the preexisting humoral and cellular immunity against common human Ad serotypes. Moreover, adenoviral particles are extremely immunogenic capsids and systemic administration of high doses of adenovirus can lead to systemic inflammatory response, which can be lethal in some extreme cases. The failure in achieving high levels of efficient transduction in target tissues is a limiting factor. Therefore, strategies have been developed for Ad modifications to enhance the efficacy while reducing dose associated toxicity^5,6^.

The specificity of Ad-mediated gene delivery depends on host cell susceptibility and permissibility for invading virus. The classical model of Ad2/5 infection primarily involves high affinity binding via the capsid fiber protein to the coxsackievirus and adenovirus receptor (CAR)^7,8^ and subsequent internalization via receptor mediated endocytosis through the capsid penton base and αv integrins^9,10^, while the *in vivo* entry pathway remains elusive. It is now widely believed that several circulating blood proteins such as coagulation factors also dictate the specificity of Ad infection^11–13^. Once the virus has successfully entered the host cells, viral DNA is subsequently released to the nuclei for DNA replication. Therefore, strategies targeting virus cell entry stages have been the primary targets to enhance Ad mediated gene delivery efficiency and specificity^14^.

It is known that viruses utilize host cell translation machinery for protein synthesis, which can be a potential target for enhanced Ad-mediated gene delivery. Adenoviruses encode a highly basic protein called protein VII that resembles cellular histones and affects cellular chromatin^15^. In the early phases of an infection, the incoming viral DNA is associated with both viral core protein VII and cellular histones. In late phases of infection, newly synthesized viral DNA is also associated with histones and tends to be wrapped in nucleosomes within the host cell nucleus^16–19^. The histone tails are subjected to modifications, including acetylation, methylation, phosphorylation, ADP ribosylation, and ubiquitination^20^. Acetylation of lysine residues within nucleosomal histone tails provides a crucial mechanism for epigenetic control of gene expression. Lysine acetylation is a reversible posttranslational modification catalyzed by histone acetyltransferases (HATs) or removed by histone deacetylases (HDACs). It is known that the adenoviral E1A protein causes significant reduction in cellular levels of lysine acetylation of histone H3^21,22^ and can disturb the normal cellular interaction between p300/CBP and its associated histone acetylase^23,24^. It is not clear how epigenetic factors may affect adenovirus replication or infection, although limited evidence suggests that histone modification is involved in the regulation of adenovirus gene expression^17,25^. For example, inhibitors of histone deacetylase (HDACi) have been demonstrated to promote both wild type Ad infection and gene delivery efficiency by replicative defective Ad viruses^19,26–28^.

Lysine acetylation alters the electrostatic properties of histones, and often creates docking sites for bromodomain-containing “reader” proteins. BRD4 is a member of the BET (bromodomain and extra-terminal domain) family protein that is involved in multiple processes of the DNA virus life cycle, including viral replication, genome maintenance, and gene transcription^29^. BRD4 serves as a ‘reader’ to recognize acetylated lysine in histones and to recruit positive transcriptional elongation factor b (P-TEFb), thereby promoting transcriptional activity and chromatin remodeling^30^. JQ1 is a BRD4 inhibitor that selectively interacts with the BD1 and BD2 domains of BRD4 and disrupts the interaction of BRD4 protein with acetylated lysine^31,32^. BRD4 has been shown to regulate HPV and HSV infection, while JQ1 treatment reactivates HIV from latency^33–35^.

In consideration of the significance of histone modifications in gene regulation, we tested whether JQ1 and structurally different BRD4 inhibitors had any effect on Ad infection and Ad-mediated gene delivery. We found that JQ1and PFI-1 promoted Ad infection and Ad-mediated EGFP gene delivery. Here we report the identification of JQ1 as a potent enhancer on Ad infection and Ad-mediated gene delivery.

## Materials and Methods

### Cells and viruses

Human lung adenocarcinoma A549 epithelial cell line (ATCC, CCL-185), human cervical carcinoma HeLa cell line (ATCC, CCL-2), human acute T cell leukemia Jurkat cell line (ATCC, TIB-152), and murine lymphocytic leukemia P388D1 cell line (ATCC, CCL-46) were purchased from ATCC (Manassas, VA) or from Cell Bank of Chinese Academy of Sciences (Shanghai, China). The cells were cultured in DMEM (high glucose) supplemented with 10% heat-inactivated fetal bovine serum (FBS), sodium pyruvate, non-essential amino acids, and penicillin-streptomycin (all from Life Technologies, Carlsbad, CA) in a humidified incubator with 5% CO_2_ at 37 °C. Wild type Ad2 was a kind gift from Dr. Glen Nemerow’s lab. The virus was propagated and titrated in A549 cells. Ad-EGFP was generated in the lab using the Ad-Easy system^36^. The viruses were CsCl gradient purified and dialyzed against Tris-HCl (pH 8.1, 10 mM) buffered saline solution and titrated by plaque forming assay following a reported protocol^37^.

### Reagents and Antibodies

PFI-1, RVX-208, LDC000067 (CDK9 inhibitor)^38^, and flavoripidol (RNA Pol II inhibitor)^39^ were purchased from Selleck Chemicals. JQ1 and (−)-JQ1 were purchased from MedChem Express. Antibodies to BRD4 (Abcam, ab128874), CDK9 (Santa Cruz, sc-13130), phospho-histone-3 (Ser10), acetyl-histone-3 (K56) (Beyoyime, Nantong), GAPDH (Bioworld Technology) were obtained commercially. The anti-CAR monoclonal antibody RmcB was from ATCC (CRL-2379). Rabbit antiserum to Ad2 hexon (GenScript, Nanjing), and to Ad2 penton base (ABmart, Shanghai) were prepared using commercial sources. Horse radish peroxidase (HRP)-conjugated secondary antibodies were purchased from Sigma-Aldrich.

### Infection assay and drug treatment

For treatment with chemicals, the compounds dissolved in DMSO were first tested on A549 cells to determine a minimal cytotoxicity concentration using an MTT method^40^. We tested JQ1 on A549 cells by treating the cells with 10, 30, 100, 300, 500 and 1000 nM JQ1 for 48 hr, using 0.1% DMSO as a vehicle control. We used 48 h since most of the infection assays lasted 24–36 hr or less. At up to 500 nM, the treatment did not significantly affect cell viability. At up to 1000 nM, (−)-JQ1, RVX-208, PFI-1 showed no toxic effect to A549 cells after 48 h treatment.

For infections, the cells were plated in culture plates for overnight. The cells were treated at 1 h prior to inoculation or at time points indicated. For immunoblotting assays, the cells were collected and lysed with a buffer containing 150 mM NaCl, 50 mM Tris-HCl (pH 7.4), 1% NP-40, and a cock-tail of protease inhibitors (Roche). The lysates were separated by SDS PAGE and transferred to PVDF membrane (Millipore) for immunoblotting analysis. GAPDH was used as a loading control. The images were captured using a ChemiScope imaging system (Shanghai, China).

For titration of infectious Ad production, the cells were collected with a cell scraper and pooled with the culture medium. The pooled samples were processed by freeze-thaw in liquid nitrogen for 3 times. The supernatants were collected by centrifugation at 5000 × *g* for 15 min and stored at −70 °C for titration analysis.

### RT-PCR and real-time PCR

For RT-PCR studies, total RNA was extracted using TRIzol reagent (Life Technologies). One microgram RNA was reverse transcribed into cDNA using AMV reverse transcriptase. Real-time PCR was performed using SYBR Green PCR Master Mix (Q141-02/03, Vazyme, Nanjing, China) on an ABI 7300 PCR system (Applied Biosystems) and data was analyzed using the 2^−ΔΔCt^ method to obtain relative abundance. GAPDH was used as an internal control for normalization. RNA levels from mock-treated samples of 3 h PI were assigned a value of 1 and used for comparison of RNA levels from corresponding samples. The primers used for hexon and fiber protein gene of Ad2 (accession number J01917) are as follows. Hexon gene: 5′-AACTTCCAGCCCATGAGC (forward) and 5′-TTGGCCCAGGTCTGTGAG (reverse). Fiber protein gene: 5′-ACCCCGTGTATCCATATGAC (forward) and 5′-ACGCGTAGAGAGAGAACTCC (reverse). GAPDH: 5′-ACAGTCAGCCGCATCTTCTT (forward) and 5′-ACGACCAAATCCGTTGACTC (reverse).

### Flow cytometry study

Flow cytometry analysis was performed on FACS Calibur (BD Bio-sciences, San Jose, CA) to determine the levels of gene expression and delivery by Ad-EGFP transduction. The percentages of GFP positive cells and the mean fluorescence intensity (MFI) values were analyzed using the preinstalled software.

### RNA interfering

The oligoes of small interfering RNA (siRNA) targeted human BRD4 were synthesized by GenePharma (Shanghai, China). For gene knockdown experiments, cells were plated in 12-well plates (Corning) 24 h before transfection. Cells were transfected using Lipofectamine-2000 and were used at 48–72 h after transfection for further experiments.

BRD4-siRNA #1: 5′-CUCCCUGAUUACUAUAAGATT and 5′-UCUUAUAGUAAUCAGGGAGTT;

BRD4-siRNA #2: 5′-GGAGAUGACAUAGUCUUAATT and 5′-UUAAGACUAUGUCAUCUCCTT;

BRD4-siRNA #3: 5′-GCACAAUCAAGUCUAAACUTT and 5′-AGUUUAGACUUGAUUGUGCTT.

### Animal study

All experimental procedures were carried out strictly in accordance with the guide for the care and use of laboratory animals and the related ethical regulations instilled at Nanjing University Medical School. Female DBA/2J Nju (N000016) mice of 4–6 weeks of age were purchased from Model Animal Research Center of Nanjing University, and were housed under pathogen-free conditions on a 12/12 h light/dark cycle with free access to food and water.

The lymphocytic leukemia P388D1were transduced with Ad-EGFP for 2 h. The cells were then collected and washed 3 times with ice-cold PBS. The cells resuspended in PBS at 1 × 10^7^ cells/ml were injected into the syngeneic DBA/2J mice peritoneally at 100 μl/mouse following a reported protocol^41^. The animals (3 per group) were then randomly grouped and then treated with a vehicle (control group), or JQ1 (3 and 10 mg/kg) by IP injection on day 2, 4, and 6 after inoculation. JQ1 freshly dissolved in DMSO was diluted with normal saline (final DMSO at approximately 1%). The ascetic cells were harvested on day 7 by rinsing the peritoneal cavity with 1.5 ml ice-cold PBS. The cells were sieved through 75-μm cell strainers, and red blood cells were removed by treatment with a red blood cell lysis buffer (Beyotime). After washing twice with PBS, the cells were resuspended in 0.5 ml ice-cold PBS and analyzed by flow cytometry for EGFP expression.

### Statistical analysis

Each experiment was performed two or more times, with three technical replicates of each culture, condition or time point in each experiment. Data from one representative experiment are presented except otherwise stated. Data were analyzed by one-way ANOVA method using SPSS 17.0 software package.

### Availability of materials and data statement

All data generated or analyzed during this study are included in this published article.

### Use and care of experimental animals

The committee at Nanjing University Medical School approved the experimental procedures for use and care of laboratory animals (mice).

## Results

### JQ1 enhances Ad infection dose-dependently

**Figure 1:**
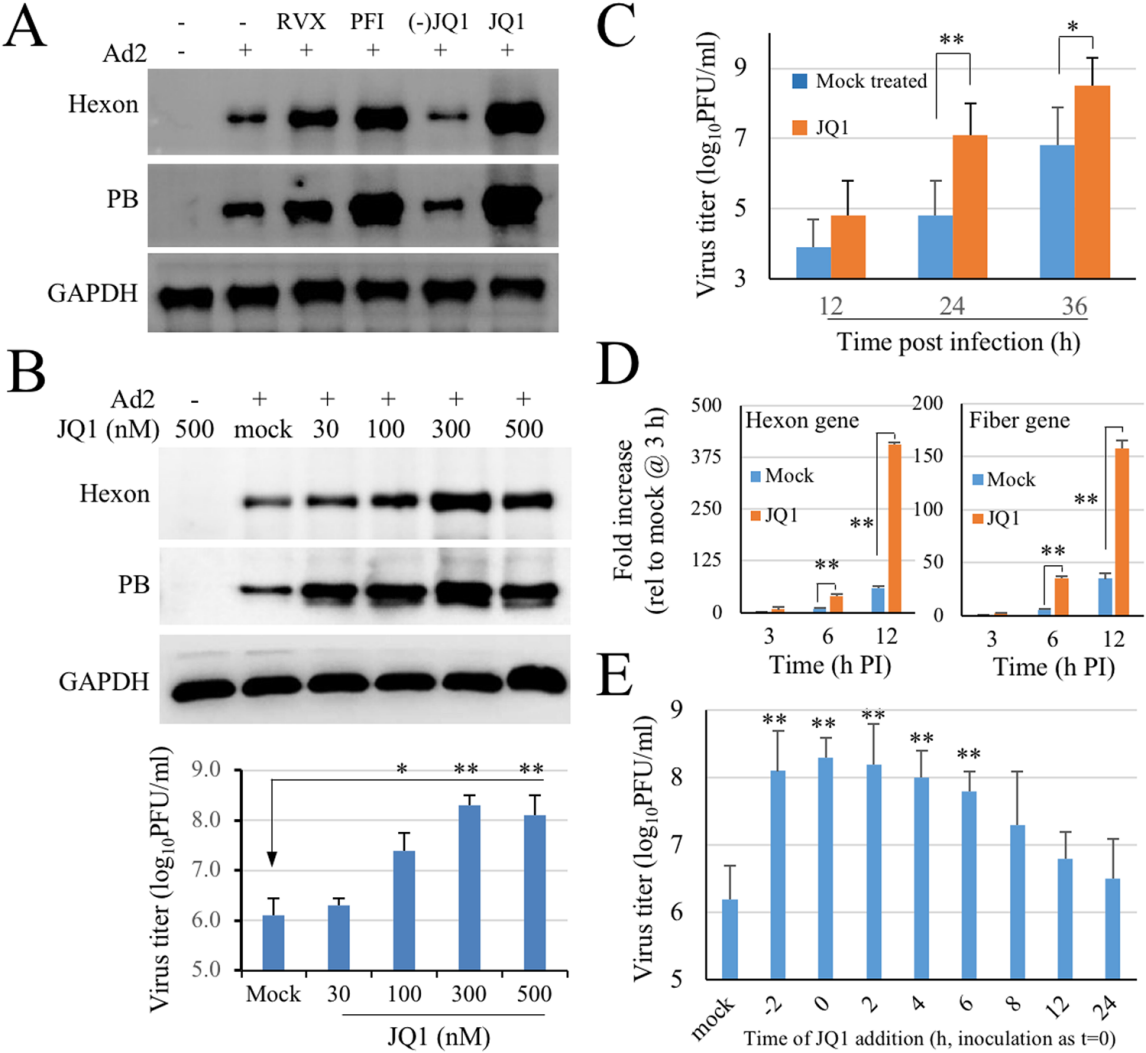
Bromodomain inhibitor JQ1 enhances adenovirus type 2 (Ad2) infection. (**A**) Effect of Bromodomain inhibitors on Ad infection. A549 cells were mock-treated (0.1% DMSO in culture medium) or treated with RVX-208 at 500 nM, PFI-1 at 500 nM, JQ1 at 300 nM, or with 300 nM (−)-JQ1, an inactive enantiomer of JQ1. The cells were then infected with Ad2 at 1 PFU/cell for 24 h. The compounds were left in the culture medium throughout the infection. Viral hexon and penton base (PB) protein was detected by immunoblotting analysis. GADPH expression was used as a loading control. The experiment was performed 3 times independently. (**B**) Dose effect of JQ1 on Ad2 infection. A549 cells were mock-treated or treated with JQ1 at indicated concentrations. The cells were infected with 0.3 PFU/cell of Ad2 an hour later. Viral protein expression was detected at 24 h post infection (PI). The experiment was performed 3 times independently. In parallel experiments (lower panel), the samples were harvested at 36 h PI for titration of virus production by plaque forming assay. The data are presented as mean ± SD of triplicate samples. *p < 0.05, **p < 0.01. (**C**) Ad2 production during JQ1 treatment. A549 cells in 24-well plates were treated with 300 nM JQ1 1 h prior to Ad2 inoculation. The samples were harvested at 12, 24 and 36 h post Ad2 inoculation and used for titration of Ad2 using duplicate samples. Titration data are presented as mean ± SD of triplicate wells, *p < 0.05, **p < 0.01. (**D**) JQ1 treatment on hexon and fiber gene expression. A549 cells were mock-treated or treated with 300 nM JQ1 at 1 h prior to Ad2 infection. Total RNA was collected and used for detection of hexon and fiber gene expression by real-time PCR. Gene expression at 3 h PI of mock-treated samples was arbitrarily assigned as 1 using GAPDH as an internal control. Mock refers to vehicle-treated and infected controls. The experiment was performed 3 times independently. **p < 0.01. (**E**) Time of JQ1 addition on Ad2 infection. A549 cells were treated with 300 nM JQ1 at 2 h prior to (-2), during (0) or at time points indicated after inoculation with 0.3 PFU/cell of Ad2. The samples were collected at 36 h PI and used for virus titration. The experiment was performed 3 times independently. Mock refers to samples from vehicle-treated and infected controls. Data are presented as mean ± SD of a representative experiment. ** p < 0.01.

To investigate whether BET bromodomain inhibition plays a role in Ad infection, A549 cells were treated with well-characterized BRD4 inhibitors 1 h prior to Ad2 infection. The inhibitors included JQ1, PFI-I, pan BD1/2 domain inhibitors, and RVX-208, a BD2 domain inhibitor. (−)-JQ1, an inactive enantiomer of JQ1, was included as a control. For a quick assay, the cells were infected with Ad2 at a MOI of 1 PFU per cell for 24 h. Viral infection was determined by detection of viral protein with immunoblotting assays. As shown in Fig. 1A, treatment with JQ-1 or PFI-1, inhibitors of pan-BD domains, resulted in significant increases in hexon and penton base (PB) production. Treatment with RVX-208, a BD2 domain selective inhibitor, also increased hexon and PB production. In contrast, treatment with (−)-JQ1 showed no enhancement effect on viral protein expression.

The effect of BRD4 inhibitors on Ad infection was further studied by treating Ad infected cells with varying amount of JQ1 since the target of this compound has been well characterized^32^. It has been reported that prolonged treatment with JQ1 at micromolar concentrations causes cell cycle arrests. We noticed that JQ1 at up to 500 nM concentrations did inhibit A549 growth after 48 h treatment (refer to M&M). We therefore used 30 to 500 nM concentrations for infection assays. As shown in Fig. 1B, JQ1 at 30 to 300 nM concentrations dose-dependently increased hexon and PB expression. In parallel experiments, JQ1 treatment also increased infectious virion production. There was an 1–2 log increase of infectious Ad in the samples treated with 100, 300, and 500 nM JQ1 (Fig. 1B). In a separate experiment, the samples were assayed at 12, 24 and 36 h post Ad infection. As shown in Fig. 1C, JQ1 treatment increased the yield of Ad production throughout the infection process. The enhancement effect of JQ1 on Ad infection was also demonstrated at mRNA levels. As shown in Fig. 1D, JQ1 treatment significantly increased hexon and fiber gene expression.

We also determined the time effect of JQ1 addition to Ad infection. In this regard, A549 cells were treated with 100 nM JQ1 prior to or during Ad2 infection. The samples were harvested at 36 h after infection for titration assay. As shown in Fig. 1E, addition of JQ1 at 2 h prior to (−2 h), during inoculation (0 h), or at 2, 4, and 6 h post Ad infection resulted in significant increase in infectious Ad2 production. The effect became less significant when JQ1 was added at 8–24 h PI.

Those results together showed that treatment with BRD4 inhibitors enhanced Ad infection.

### JQ1 enhances Ad-mediated gene delivery

**Figure 2:**
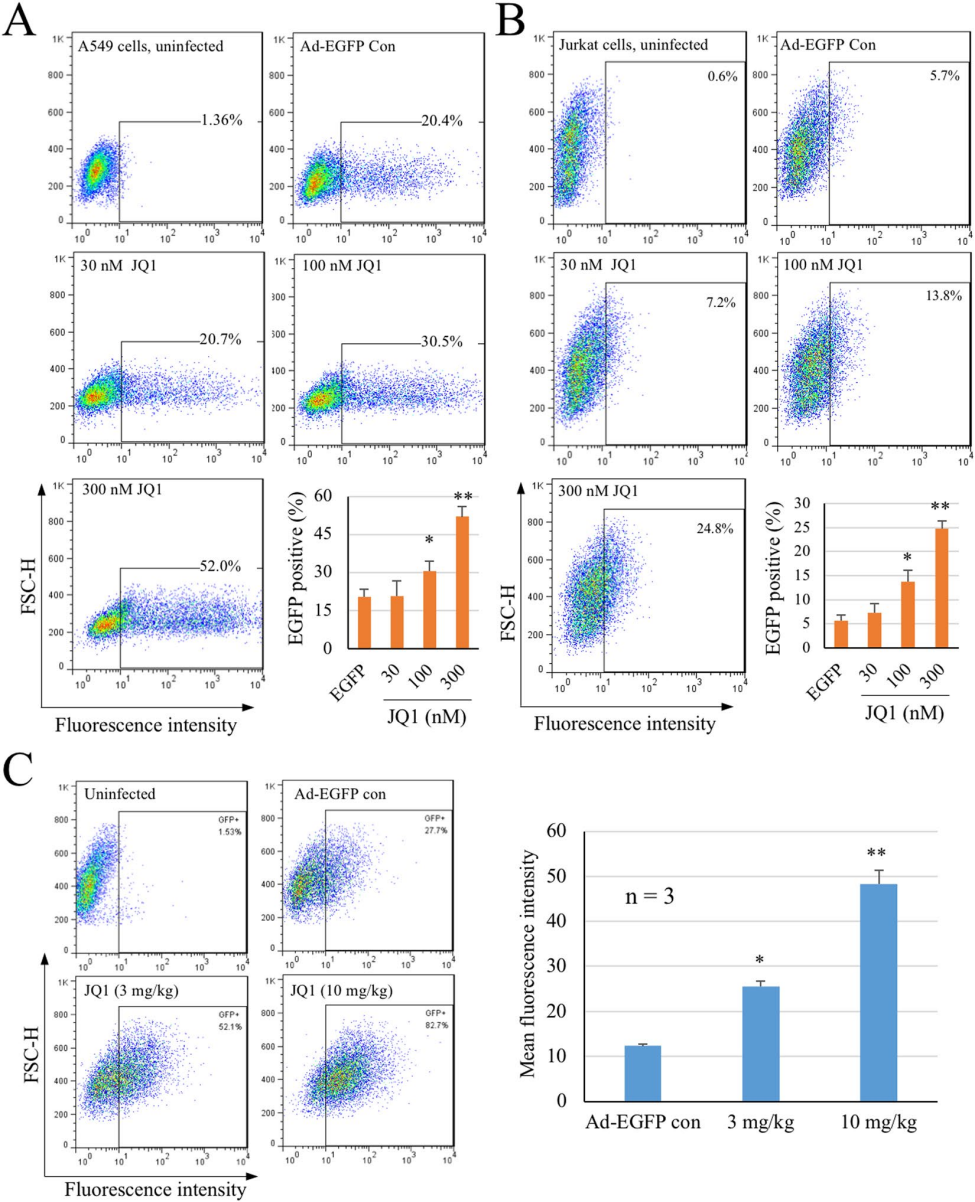
JQ1 enhances EGFP expression delivered by an adenoviral vector. (**A**,**B**) JQ1 promotes EGFP expression in cell cultures. A549 or Jurkat cells were uninfected or infected with Ad-EGFP in the absence or presence of varying amounts of JQ1 for 24 h. The cells were harvested and EGFP expression was measured by flow cytometry analysis. The bar graphs represent quantitative measurement of EGFP-positive cell population of triplicate samples. Data represent mean ± SD. *p < 0.05, **p < 0.01. (**C**) JQ1 promotes EGFP expression in a murine tumor model. Mouse P388D1 cells were uninfected or infected with Ad-EGFP for 2 h. After washing with serum-free DMEM, the cells (1 × 10^6^ cells/mouse) were implanted in the syngeneic DBA2 mouse by intraperitoneal route. The animals were then treated with JQ1 at 3 and 10 mg/kg by IP injection on day 2, 4, and 6 after P388D1 implantation. The cells were collected on day 7 and analyzed for EGFP-positive cells by FACS analysis. The experiment was performed twice independently. Data are presented as mean ± SD of mean fluorescence intensity of one experiment (MFI, n = 3). *p < 0.05, **p < 0.01.

To expand these findings, we investigated whether JQ1 treatment affected Ad-mediated gene delivery using a replicative defective Ad. In this regard, A549 epithelial cells or Jurkat T lymphocytes were infected with an E1 deleted Ad5 that encodes an EGFP reporter gene (Ad-EGFP). The cells were then treated with JQ1 or remained untreated. EGFP expression was quantitatively determined by FACS analysis 24 h PI. As shown in Fig. 2A and B, JQ1 treatment increased EGFP expression. At 100 and 300 nM, JQ1 markedly increased the population of EGFP-expressing cells.

The findings were verified using an implanted tumor model^42^. To this end, the mouse leukemia P388D1 cells were infected with Ad-EGFP for 2 h. After washing with PBS, the infected cells or mock-treated control cells were resuspended in PBS and injected intraperitoneally to the syngeneic DBA2 mice. The animals were treated with JQ1 at 3 and 10 mg/kg on the 2^rd^, 4^th^, and 6^th^ day post P388D1 inoculation, or mock-treated of the uninfected controls. On the 7^th^ day, the cells from the peritoneal cavity were collected by rinse with PBS and EGFP expression was measured by flow cytometry. As shown in Fig. 2C, treatment with JQ1 at 3 and 10 mg/kg significantly increased EGFP expression compared with infected but mock-treated controls (Ad-EGFP con, p < 0.01). The results together showed that JQ1 treatment enhanced Ad-mediated gene delivery.

### Requirement of BRD4 in Ad infection and JQ1 effect on Ad infection

**Figure 3:**
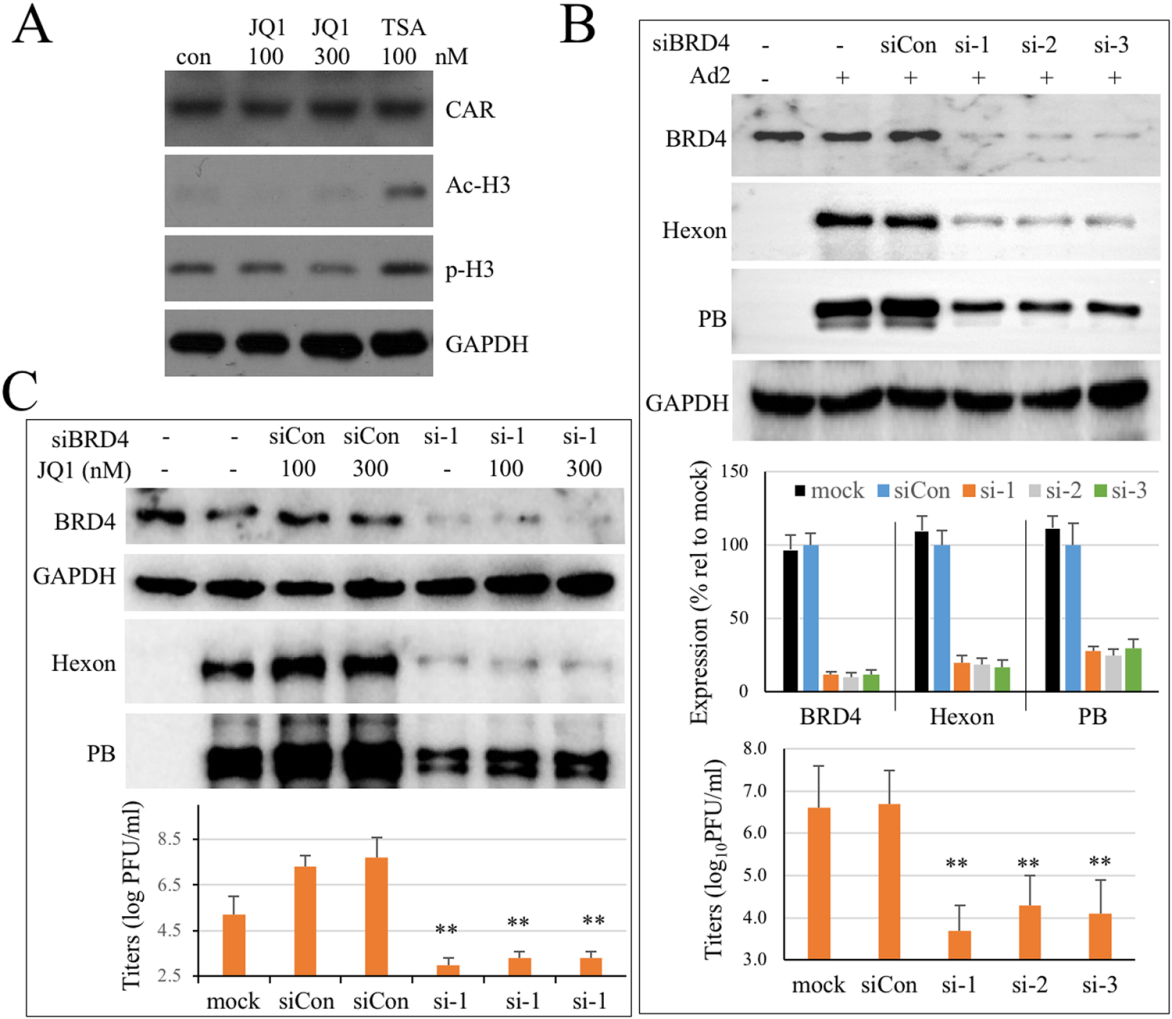
Requirement of BRD4 in Ad2 infection and JQ1 enhancement effect. (**A**) JQ1 treatment does not affect CAR expression nor histone-3 modifications. A549 cells were treated with JQ1 at 100 or 300 nM, or treated with trichostatin A (TSA) at 100 nM for 24 hr. The cells were lysed and CAR expression or histone-3 modifications were determined by immunoblotting analysis. The experiment was performed 2 times independently. (**B**) Knock down of BRD4 expression suppresses viral protein expression and Ad2 infection. A549 cells were treated with a scrambled siRNA (siCon) or with 3 different siRNA that were reported to suppress BRD4 expression. The cells were infected with Ad2 (MOI = 1) at 60 h post siRNA transfection. BRD4 expression and viral protein production was determined 24 h after Ad2 infection. Upper panel, siBRD4 effect on BRD4 expression and hexon and PB production. The bar graph represents the effect from 3 independent experiments using siCon as controls for corresponding proteins. In separate experiments, suppression of BRD4 expression on Ad2 infection was validated by titration for infectious Ad2 production using triplicate samples. Mock refers to infected but untreated controls. **p < 0.01. (**C**) Knock down of BRD4 expression blocks JQ1 enhancement effect on Ad2 infection. A549 cells were transfected with siCon or with siRNA-1 targeting BRD4 expression. Sixty hours later, the cells were infected with Ad2 (0.3 PFU/cell) in the absence or presence of JQ1 at indicated concentrations. BRD4 and viral protein expression was determined by immunoblotting assay. In parallel experiments, the samples were harvested at 36 h PI for virus titration. Mock refers to infected but untreated controls. **p < 0.01.

HDAC inhibitors like FK228 and trichostatin A (TSA) were reported to increase Ad infection by promoting CAR expression^43,44^. We first investigated whether JQ-1 treatment altered CAR expression by immunoblotting for CAR expression. A549 cells expresses abundant CAR protein. JQ1 at 100 and 300 nM did not significantly affect CAR expression. Unlike TSA that caused histone-3 acetylation, JQ1 treatment did not cause histone-3 modifications (Fig. 3A).

JQ1 was originally developed as a specific BRD4 inhibitor that selectively interacts with the BD1 and BD2 domains and disrupts BRD4 interaction with acetylated lysine residues. We asked whether JQ1 promoted Ad infection through BRD4 protein since Brd4 release from chromatin is essential for its role transition from chromatin targeting to transcriptional regulation^45^. A549 cells were therefore treated with siRNA to suppress BRD4 expression. We identified 3 different siRNA that suppressed BRD4 expression effectively (Fig. 3B). Suppression of BRD4 expression not only blocked Ad infection but the JQ1 effect on Ad2 infection (Fig. 3C). The results suggested to us that both Ad infection and JQ1 effect on the infection required BRD4 protein expression.

### Ad infection promotes BRD4 association with CDK9

**Figure 4:**
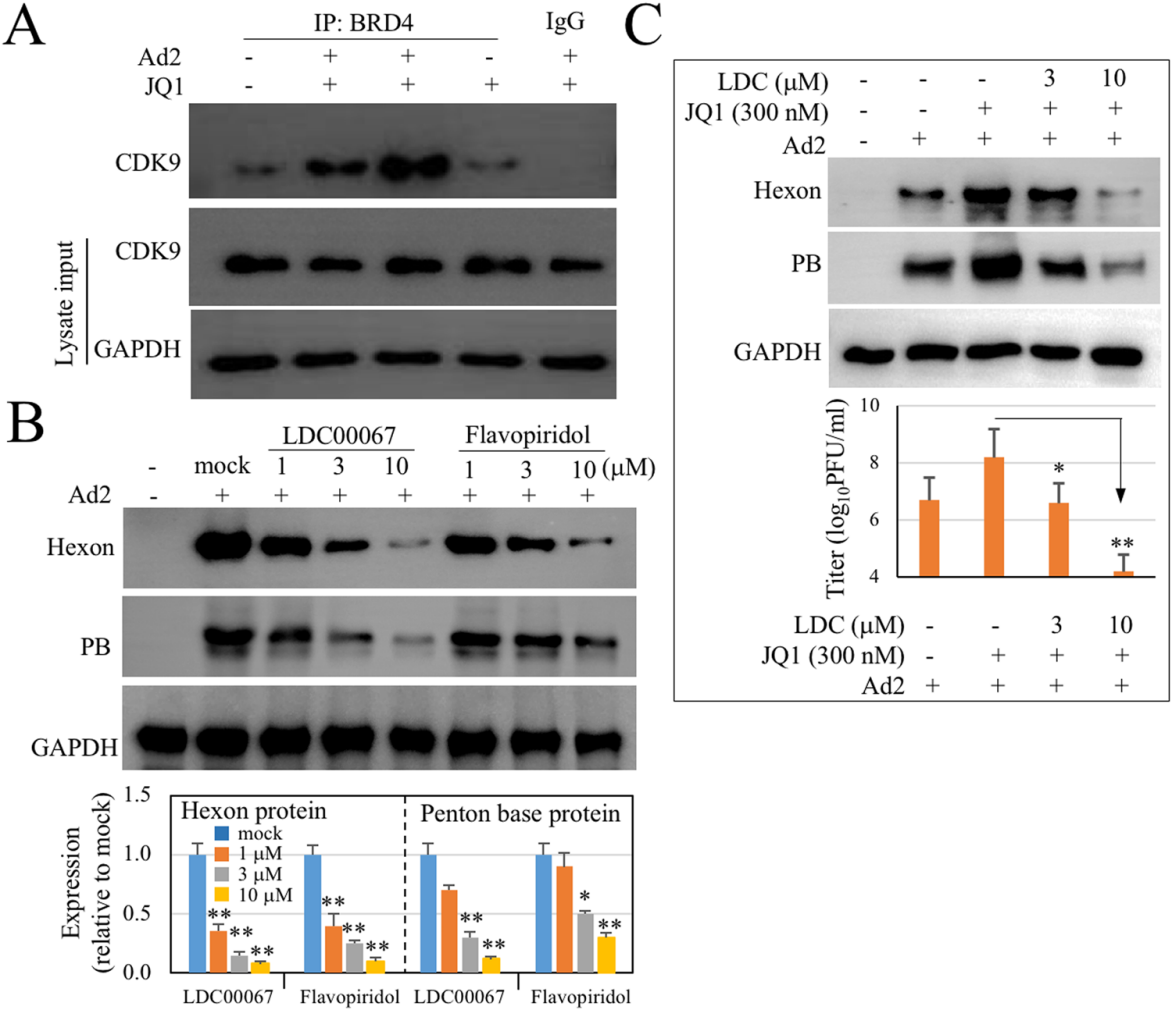
JQ1 enhances Ad infection through BRD4 and CDK9 association. (**A**) Ad2 infection induces BRD4 and CKD9 association that is enhanced by JQ1 treatment. A549 cells were infected with Ad2 for 5 h in the absence or presence of 300 nM JQ1. CDK9, a subunit of P-TEFb, in the anti-BRD4 immunocomplex was detected by immunoblotting. Input denotes proteins in the whole cell lysates. (**B**) Inhibition of Ad protein synthesis by CDK9 inhibitors. A549 cells were infected with Ad2 in the absence or presence of inhibitors at concentrations indicated. Viral protein production was detected by immunoblotting assays. Lower panel represents the inhibitory effect on hexon and PB production of 3 independent experiments, using the ration of corresponding protein expression to GAPDH in mock-treated samples as 1. Data represent mean ± SD. *p < 0.05, **p < 0.01. (**C**) Inhibition of JQ1 enhancement effect on Ad2 infection by CDK9 inhibitor. A549 cells were infected with Ad2 in the absence or presence of 300 nM JQ1. For inhibition, LDC00067 was added simultaneously. Viral protein synthesis or virion production was detected at 24 and 36 h PI, respectively. The experiments were performed 2 times independently. Data represent mean ± SD of triplicate samples. *p < 0.05, **p < 0.01.

BRD4 actively regulates gene transcription by recruiting positive transcription elongation factor b (P-TEFb) to gene promoters^46,47^. We tested whether JQ1 promoted Ad infection by through gene transcription. In this regard, we examined BRD4 association with P-TEFb. A549 cells were infected with Ad2 (10 PFU/cell) in the absence or presence of JQ1 for 5 h. The cells were then lysed and cell lysates were immunoprecipitated with anti-BRD4 antibody. P-TEFb and BRD4 association was detected by immunoblotting for CDK9, a subunit of P-TEFb, in the complexes. As shown in Fig. 4A, BRD4 and CDK9 had weak association in mock-infected samples. Ad infection resulted in increased detection of CDK9 in the anti-BRD4 immunocomplexes. Concurrently with an increase in Ad infection, BRD4-CDK9 association was also increased in Ad-infected and JQ1-treated samples. These results indicated that JQ1 promoted BRD4 and P-TEFb transcription complex formation.

To corroborate this conclusion, we first determined whether Ad infection required CDK9 activity by treating the cells with CDK9 inhibitors 1 h prior to infection. As shown in Fig. 4B, pretreatment of A549 cells with LDC000067, a selective inhibitor of CDK9 activity^38^, or with flavopiridol, an inhibitor of P-TEFb activity and subsequent RNA Pol II transcription^39^, significantly reduced hexon and PB protein synthesis. In addition, we found that LDC00067 treatment also blocked JQ1 enhancement effect on Ad infection (Fig. 4C).

Together, the study demonstrated that Ad infection required BRD4 expression. The study also showed that BRD4 inhibition can potentiate Ad-mediated gene delivery efficiency.

## Discussion

We found that BRD4 inhibitors like JQ1 promoted Ad infection and Ad-mediated gene delivery. JQ1 is an inhibitor that blocks acetylated lysine interaction with the bromodomains of BRD4 and related proteins. As a multiple domain protein involved in cell regulation, BRD4 plays a critical role in gene transcription. We found that Ad infection induced BRD4 and CDK9 association, an event that was exacerbated by JQ1 treatment, suggesting that JQ1 likely regulates transgene expression by gene transcription. The conclusion was demonstrated using Ad2 and Ad5-based vectors, it is likely that the mechanism may apply to other types of adenoviruses since CDK9 inhibitors have been reported to inhibit replication of multiple DNA viruses^35,48^. This study therefore uncovers a pathway involved in Ad infection and Ad-mediated gene expression.

To establish infection in the host cell, adenovirus must rapidly express its early genes which are responsible for reconfiguring the cellular pathways and checkpoints, thus modifying the environment for optimal viral replication. Adenoviruses, like other DNA viruses, exist as nuclear episomes and are very likely to exploit epigenetic mechanisms to regulate biological activities during their life cycle^21,49,50^. Although the Ad2 E1A protein has a global effect on cellular transcription by directly impact on histone modifications^22^ and the activation of a subset of IFN-stimulated genes (ISGs)^51^, the assembly of a helper-dependent adenovirus DNA into chromatin for efficient gene expression is independent of viral DNA replication^25,52^. Similarly, we found that JQ1 also promoted gene delivery efficiency by an E1-deleted Ad, indicating transgene expression is independent of E1 gene function. We found that suppression of BRD4 expression or inhibition of P-TEFb activity blocked Ad infection, indicating that Ad exploits gene transcription mechanisms for productive infection and transgene expression.

BRD4 is a scaffold protein that recruits P-TEFb for gene transcription via increased Pol II phosphorylation by CDK9 subunit of P-TEFb. Notably, BRD4 interacts with P-TEFb via its P-TEFb interaction domain (PID), thereby stimulating its kinase activity and stimulating its phosphorylation of the carboxyl-terminal domain (CTD) of RNA polymerase II for transcription elongation^53,54^. BRD4 has been shown to regulate virus infection with diverse mechanisms^55^. It interacts with papillomavirus E2 protein and tethers the viral DNA to host mitotic chromosomes for persistence of viral episomes in HPV-infected cells^56^; whereas the BRD4 PID suppresses the ability of HIV Tat transactivator by disrupting the interaction between the Tat and P-TEFb^33^. We showed here that BRD4 interacts with CDK9 and regulates Ad infection and Ad-mediated gene delivery. Further studies are required to delineate the mechanisms by which BRD4 utilizes to regulate adenovirus infection and transgene expression.

The Ad DNA is wrapped in nucleosomes and, as with cellular chromatin, changes in the epigenetic status of the histones impact upon regulation and expression of Ad encoded transgenes^25^. FK228, TSA, and butyrate, HDAC inhibitors, have been known to enhance Ad-mediated gene delivery efficiency. It was thought that FK228 and TSA enhanced Ad transduction through upregulation of the attachment receptor CAR^43,57^. However, the highest increase in transgene expression was achieved when non-toxic concentrations of FK228 were added immediately after transduction and was associated with increased histone H3 acetylation^27,28^, suggesting that FK228 works mainly by increasing transgene expression at the transcriptional levels. One of the major obstacles to success in gene therapy is transcriptional silencing of the DNA vector. The mechanisms underlying gene silencing/repression in mammalian cells are complex, but changes in chromatin structure and, in particular, histone modifications have been identified as a major factor in transcriptional regulation of endogenous genes. Those and our studies demonstrate epigenetic regulation pathways may be exploited for enhanced gene expression and transduction.

Adenovirus vectors have a number of advantages, including the adenovirus genome is well characterized and easy to manipulate and to produce to high purity, to name a few. In fact, among the almost 2,600 gene therapy clinical trials approved worldwide, a significant portion of the trials have been utilizing adenoviral vectors^2^. The nature that adenoviruses can drive robust and long-lasting immune responses of CD8 has led to vaccine designs using Ad vectors against pathogens like H5N1, Ebola, and Zika viruses^58^. The demonstration of JQ1 enhances Ad infection and Ad-mediated gene delivery may result in fewer vector doses for necessary gene expression, helping to alleviate disadvantages caused by Ad vectors for gene and cell-based therapies in the new era of personalized medicine.
